# Methylsulfonylmethane ameliorates metabolic-associated fatty liver disease by restoring autophagy flux via AMPK/mTOR/ULK1 signaling pathway

**DOI:** 10.3389/fphar.2023.1302227

**Published:** 2023-11-30

**Authors:** Daewon Han, Deokryong Kim, Haeil Kim, Jeonga Lee, Jungmook Lyu, Jong-Seok Kim, Jongdae Shin, Jeong Sig Kim, Do Kyung Kim, Hwan-Woo Park

**Affiliations:** ^1^ Department of Cell Biology, Konyang University College of Medicine, Daejeon, Republic of Korea; ^2^ Myunggok Medical Research Institute, Konyang University College of Medicine, Daejeon, Republic of Korea; ^3^ Department of Medical Science, Konyang University, Daejeon, Republic of Korea; ^4^ Department of Obstetrics and Gynecology, Soonchunhyang University Seoul Hospital, Seoul, Republic of Korea; ^5^ Department of Anatomy, Konyang University College of Medicine, Daejeon, Republic of Korea

**Keywords:** methylsulfonylmethane, obesity, autophagy, AMPK/mTOR, ULK1

## Abstract

**Introduction:** Metabolism-associated fatty liver disease (MAFLD) is a global health concern because of its association with obesity, insulin resistance, and other metabolic abnormalities. Methylsulfonylmethane (MSM), an organic sulfur compound found in various plants and animals, exerts antioxidant and anti-inflammatory effects. Here, we aimed to assess the anti-obesity activity and autophagy-related mechanisms of Methylsulfonylmethane.

**Method:** Human hepatoma (HepG2) cells treated with palmitic acid (PA) were used to examine the effects of MSM on autophagic clearance. To evaluate the anti-obesity effect of MSM, male C57/BL6 mice were fed a high-fat diet (HFD; 60% calories) and administered an oral dose of MSM (200 or 400 mg/kg/day). Moreover, we investigated the AMP-activated protein kinase (AMPK)/mechanistic target of rapamycin complex 1 (mTORC1)/UNC-51-like autophagy-activating kinase 1 (ULK1) signaling pathway to further determine the underlying action mechanism of MSM.

**Results:** Methylsulfonylmethane treatment significantly mitigated PA-induced protein aggregation in human hepatoma HepG2 cells. Additionally, Methylsulfonylmethane treatment reversed the PA-induced impairment of autophagic flux. Methylsulfonylmethane also enhanced the insulin sensitivity and significantly suppressed the HFD-induced obesity and hepatic steatosis in mice. Western blotting revealed that Methylsulfonylmethane improved ubiquitinated protein clearance in HFD-induced fatty liver. Remarkably, Methylsulfonylmethane promoted the activation of AMPK and ULK1 and inhibited mTOR activity.

**Conclusion:** Our study suggests that MSM ameliorates hepatic steatosis by enhancing the autophagic flux via an AMPK/mTOR/ULK1-dependent signaling pathway. These findings highlight the therapeutic potential of MSM for obesity-related MAFLD treatment.

## 1 Introduction

Metabolism-associated fatty liver disease (MAFLD), characterized by excessive accumulation of fat in the liver associated with obesity and insulin resistance, is a serious global health concern (Diehl and Day, 2017). MAFLD encompasses several conditions, including metabolic-associated steatohepatitis, advanced liver fibrosis, cirrhosis, and hepatocellular carcinoma, and is closely associated with various risk factors, such as obesity, type 2 diabetes mellitus, cardiovascular complications, and metabolic syndrome (Loomba et al., 2020; Sheka et al., 2020). The strong association between obesity and MAFLD suggests obesity-associated inflammation and insulin resistance as key factors that trigger or worsen this condition (Marra and Svegliati-Baroni, 2018). Lipotoxicity, oxidative stress, inflammation, genetics, and metabolism play important roles in the pathogenesis of MAFLD (Park et al., 2014a; Cho et al., 2018; Marra and Svegliati-Baroni, 2018); however, the exact mechanism underlying MAFLD development remains elusive.

Autophagy is a conserved catabolic process that is crucial for degrading and recycling unnecessary intracellular components, such as excessive lipid droplets, misfolded protein aggregates, and damaged organelles, to maintain the liver metabolic homeostasis (Klionsky and Emr, 2000; Khambu et al., 2018). Impaired autophagy hinders the clearance of excess lipids from lipid droplets, inclusion bodies, and toxic protein aggregates, thereby contributing to MAFLD development (Park et al., 2014b; Park and Lee, 2014; Zhang et al., 2018). Sequestosome-1 (p62/SQSTM1; hereafter referred to as p62) is an autophagy adaptor protein responsible for autophagic degradation of polyubiquitinated substrates (Pankiv et al., 2007). p62 binds to polyubiquitinated substrates via its ubiquitin-associated domain and interacts with microtubule-associated protein light chain 3 (LC3) on the autophagosome (Bjorkoy et al., 2009; Isogai et al., 2011). Impaired autophagy contributes to several pathological conditions, including fatty liver disease and hepatocellular carcinoma progression (Mizushima and Klionsky, 2007; Niture et al., 2021).

Recent studies are focusing on identifying natural compounds that are relatively safe and have potentially beneficial effects in obesity-related diseases. Methylsulfonylmethane (MSM) is an organosulfur compound found in various plant and animal tissues that has been investigated for its potential biological benefits, including its anti-inflammatory, antioxidant, and analgesic effects (Kim et al., 2009; Nakhostin-Roohi et al., 2011; Ahn et al., 2015). MSM exerts beneficial effects on obesity-induced hepatic steatosis and insulin resistance (Sousa-Lima et al., 2016; Miller et al., 2021). However, the action mechanism of MSM in metabolic syndrome remains unknown. Therefore, in this study, we aimed to explore the role of autophagy in the treatment of MAFLD with MSM.

In this study, we investigated the potential effects of MSM treatment on autophagic clearance in vitro models of obesity using palmitic acid (PA)-treated human hepatoma (HepG2) cell lines and diet-induced obese mice. Furthermore, the molecular mechanisms by which MSM regulates autophagy were studied. We observed that MSM reduced obesity-induced insulin resistance and hepatic steatosis. Mechanistically, MSM treatment markedly enhanced autophagic flux, subsequently inhibiting the accumulation of protein inclusions via the AMP-activated protein kinase (AMPK)/mechanistic target of rapamycin kinase (mTOR)/UNC-51-like autophagy-activating kinase 1 (ULK1) signaling pathway. Our findings provide a better understanding of the role of autophagy in the pharmacological treatment of MAFLD and its associated metabolic disorders using MSM-based medicine.

## 2 Materials and methods

### 2.1 Materials

MSM, fatty acid-free bovine serum albumin (BSA), and PA were purchased from Sigma-Aldrich (MO, United States). Torin 1 was purchased from Cayman Chemical (MI, United States). Bafilomycin A1, Rapamycin and AICAR were purchased from LC Laboratories (MA, United States). Compound C was purchased from Tokyo Chemical Industry (Japan). Ubiquitin (sc-8017) and ribosomal protein S6 (sc-74459) were purchased from Santa Cruz Biotechnology (CA, United States). LC-3 (2775), p62 (5114), phospho-ULK1 (5869), ULK1 (8054), phospho-AMPK (2535), AMPK (2532), phospho-p70S6K (9205), p70S6K (9202), phospho-ribosomal protein S6 (2211), and TSC2 (4308) were purchased from Cell Signaling Technology (MA, United States). β-actin (JLA20) and α-Tubulin (12G10) were purchased from Developmental Studies Hybridoma Bank (IA, United States). GAPDH (OAEA00006) was purchased from Aviva Systems Biology (CA, United States).

### 2.2 Cell culture and treatment

Human hepatoma (HepG2) cells were cultured in Dulbecco’s Modified Eagle Medium (Welgene, South Korea) supplemented with 10% fetal bovine serum (Welgene) and 100 U/mL penicillin–streptomycin (Welgene) at 37°C and 5% CO_2_. For PA treatment, the cells were incubated for the indicated time points with 500 μM PA, as previously described (Lee et al., 2019). Control cell cultures containing fatty acid-free bovine serum albumin (Sigma-Aldrich) as a vehicle were incubated along with the treated cultures.

### 2.3 Animal experiments

Eight-week-old male C57BL/6 mice were purchased from Samtako (Osan, South Korea). The mice were kept in a controlled environment at a temperature of 21°C–24 °C and humidity of 50% ± 5%. They followed a 12-h light/dark cycle and had *ad libitim* access to water and a standard rodent low-fat diet (LFD) or high-fat diet (HFD; containing 60% of calories from fat, Research Diets, New Brunswick, NJ, United States of America). MSM (200 mg/kg body weight or 400 mg/kg body weight) was administered via daily oral gavage for 4 weeks after 9 weeks of HFD intake. The mice were euthanized by CO_2_ asphyxiation and their blood samples, liver tissues, and epididymal white adipose tissues were harvested, stored at −80°C, and then used for further molecular analysis.

### 2.4 Cell viability

Cell viability was measured using a water-soluble tetrazolium salt (WST)-8 cell counting kit (catalog no. QM2500; Daeil Lab Service, South Korea), according to the manufacturer’s instructions. Briefly, HepG2 cells were plated at a final density of 1 × 10^4^ cells/well in a 96-well plate. After 24 h of incubation, the cells were treated with MSM or phosphate-buffered saline (PBS; vehicle) as indicated. Subsequently, 10 μL of WST-8 solution was added to each well and incubated for 30 min in a humidified atmosphere (37°C and 5% CO_2_). Absorbance was measured at 450 nm using an Epoch2 microplate reader (Bio-Tek Instruments, Winooski, VT, United States). Absorbance values of treated cells were expressed as a percentage of the absorbance value of the control.

### 2.5 Solubility fractionation

Solubility fractionation was performed as previously described (Kim et al., 2023). Liver tissues and HepG2 cells were washed and lysed in a lysis buffer containing 20 mM Tris-Cl pH 7.5, 150 mM NaCl, 1 mM EDTA, 1 mM EGTA, 2.5 mM NaPPi, 1 mM β-glycerophosphate, 1 mM Na_3_VO_4_, 1% Triton X-100, and a protease inhibitor cocktail. After 30 min of incubation on ice, lysates were centrifuged at 12,000 × *g* for 15 min at 4°C. The supernatant was collected as the Triton X-100-soluble fraction. The pellets were resuspended again in a lysis buffer containing 2% sodium dodecyl sulfate (Triton X-100-insoluble fraction). Samples were boiled in Laemmli’s sample buffer and subjected to sodium dodecyl sulfate-polyacrylamide gel electrophoresis (SDS-PAGE) and immunoblotting.

### 2.6 Glucose and insulin tolerance tests

For the glucose tolerance test (GTT), the mice were fasted for 6 h and intraperitoneally injected with D-glucose (1 g/kg body weight). For the insulin tolerance test (ITT), mice were fasted for 6 h and intraperitoneally injected with insulin (0.65 U/kg body weight). At the specified time intervals, blood samples were collected from the tail, and blood glucose levels were assessed using an Accu-Chek glucometer (Roche Diagnostics, Germany).

### 2.7 Biochemical measurements

Blood samples were collected and left to coagulate for 30 min at room temperature (22°C–26°C). Subsequently, the samples were centrifuged at 1,000 × *g* for 15 min to collect the serum. Serum alanine aminotransferase (ALT), aspartate aminotransferase (AST), and alkaline phosphatase (ALP) levels were determined using assay kits (ALT, catalog no. K752-100; AST, catalog no. K753-100; ALP, catalog no. K412-500; BioVision, United States).

### 2.8 Western blotting

Frozen liver tissues and HepG2 cells were homogenized on ice in a radioimmunoprecipitation assay buffer containing a cOmplete™ EDTA-free protease inhibitor cocktail (Roche, Switzerland). Lysates were then sonicated and centrifuged at 18,000 × *g* for 15 min at 4 °C to remove the cell debris. Total protein concentration was determined using bicinchoninic acid protein (Thermo Fisher Scientific, United States). Laemmli sample buffer was added to the samples and the mixture was boiled for 5 min. Proteins were separated via SDS-PAGE and transferred onto polyvinylidene fluoride membranes (Merck Millipore, Germany). Membranes were incubated with the appropriate primary antibodies against ubiquitin, p62, phospho-ULK1, ULK1, LC-3, phospho-AMPK, AMPK, phospho-p70S6K, p70S6K, phospho-ribosomal protein S6, ribosomal protein S6, TSC2, and β-actin in TBS containing 5% non-fat milk powder or BSA for 1 h at room temperature. The membranes were then washed thrice and incubated with the appropriate horseradish peroxidase-conjugated secondary antibodies. The membranes were again washed thrice and the protein bands were detected via enhanced chemiluminescence assay using the Clarity ECL Western blotting Substrate (Bio-Rad, United States). Band densities were quantified using the ImageJ software (National Institutes of Health, United States).

### 2.9 Plasmids and virus production

HEK293T cells were co-transfected with the following lentiviral and packaging vectors using a polyethyleneimine reagent: sh-Luciferase (sh-Luc), sh-ULK1, and sh-TSC2 (kindly provided by Andrei V. Budanov, Trinity College Dublin) (Park et al., 2014a). Lentiviral supernatants were collected 48 and 72 h after transfection and further concentrated using a Lenti-X concentrator (Takara, Japan), according to the manufacturer’s protocol. HepG2 cells were transduced with the concentrated lentivirus in the presence of 4 μg/mL polybrene.

### 2.10 Immunocytochemistry

HepG2 cells were fixed with 4% paraformaldehyde (pH 7.4) for 15 min at room temperature, washed thrice with PBS, and permeabilized with methanol at −20°C. The cells were incubated for 1 h in a blocking solution with a combination of anti-p62 (1:400), anti-ubiquitin (1:400), anti-LC3B (1:400), and anti-lysosomal-associated membrane protein 1 (LAMP1, 1:400) antibodies at 4°C in a humidified chamber. The cells were again washed thrice with PBS and incubated with the appropriate combination of secondary antibodies (1: 400, Alexa Fluor 488-conjugated goat anti-mouse or anti-rabbit and Alexa Fluor 549-conjugated goat anti-mouse or anti-rabbit secondary antibodies; Thermo Fisher Scientific) for 1.5 h. Nuclei were counterstained with 1 μg/mL Hoechst 33342 (Thermo Fisher Scientific) and mounted on slides using the ProLong Gold antifade reagent (Thermo Fisher Scientific). Fluorescent images were obtained using a laser scanning confocal microscope (LSM 700; Carl Zeiss, Germany).

### 2.11 Histological analysis

Liver tissues were prepared for histological examination and stained with hematoxylin and eosin (H&E; Sigma-Aldrich) and Oil red O (Sigma-Aldrich) as previously described (Lee et al., 2019). The tissues were fixed with 10% neutral buffered formalin, embedded in paraffin, and stained with H&E. Paraffin-embedded sections were deparaffinized, rehydrated, and subjected to heat-induced epitope retrieval using a citrate buffer. Endogenous peroxidase activity was blocked by incubating the tissue sections with 3% hydrogen peroxide. The sections were incubated with a blocking solution for 60 min at room temperature. Subsequently, sections were incubated overnight at 4°C with the primary antibody against anti-p62. The sections were then washed thrice with PBS and incubated with a biotinylated anti-rabbit secondary antibody (Vector Laboratories, Burlingame, CA, United States of America). The antibodies were detected using streptavidin-horseradish peroxidase (BD Biosciences) and 3,3′-diaminobenzidine (Sigma-Aldrich). The sections were counterstained with hematoxylin. Frozen liver tissues embedded in OCT were sectioned and stained with Oil red O (Sigma-Aldrich). All samples were examined under a light microscope equipped with a digital camera (Leica, Germany).

### 2.12 Reverse transcription-quantitative polymerase chain reaction (RT-qPCR)

Total RNA was extracted from the liver tissue homogenates and HepG2 cells using the TRIzol reagent (Takara), following the manufacturer’s instructions. Total RNA was reverse-transcribed into complementary DNA using a cDNA synthesis kit (catalog no. BR123; BioFact, Korea). To determine the relative gene expression, real-time PCR was performed using the SYBR Green qPCR Master Mix (BioFact) on a real-time PCR system (Life Technologies, United States). Relative fold changes in the expression of target genes were calculated using the comparative threshold cycle (Ct) method with cyclophilin A as an internal control gene to normalize the target gene expression levels. Primers used in this study are listed in Supplementary Table S1.

### 2.13 Statistical analysis

The results are presented as mean ± standard error of the mean. Unless otherwise indicated, data depicted in the figures represent at least three independent experiments. The significance of the differences between two experimental groups was determined using a two-tailed Student’s t-test. Multiple comparisons were conducted using one-way analysis of variance, followed by Tukey’s *post hoc* test. Differences among means were considered statistically significant at *p* < 0.05.

## 3 Results

### 3.1 MSM inhibits PA-induced accumulation of protein inclusions

**FIGURE 1:**
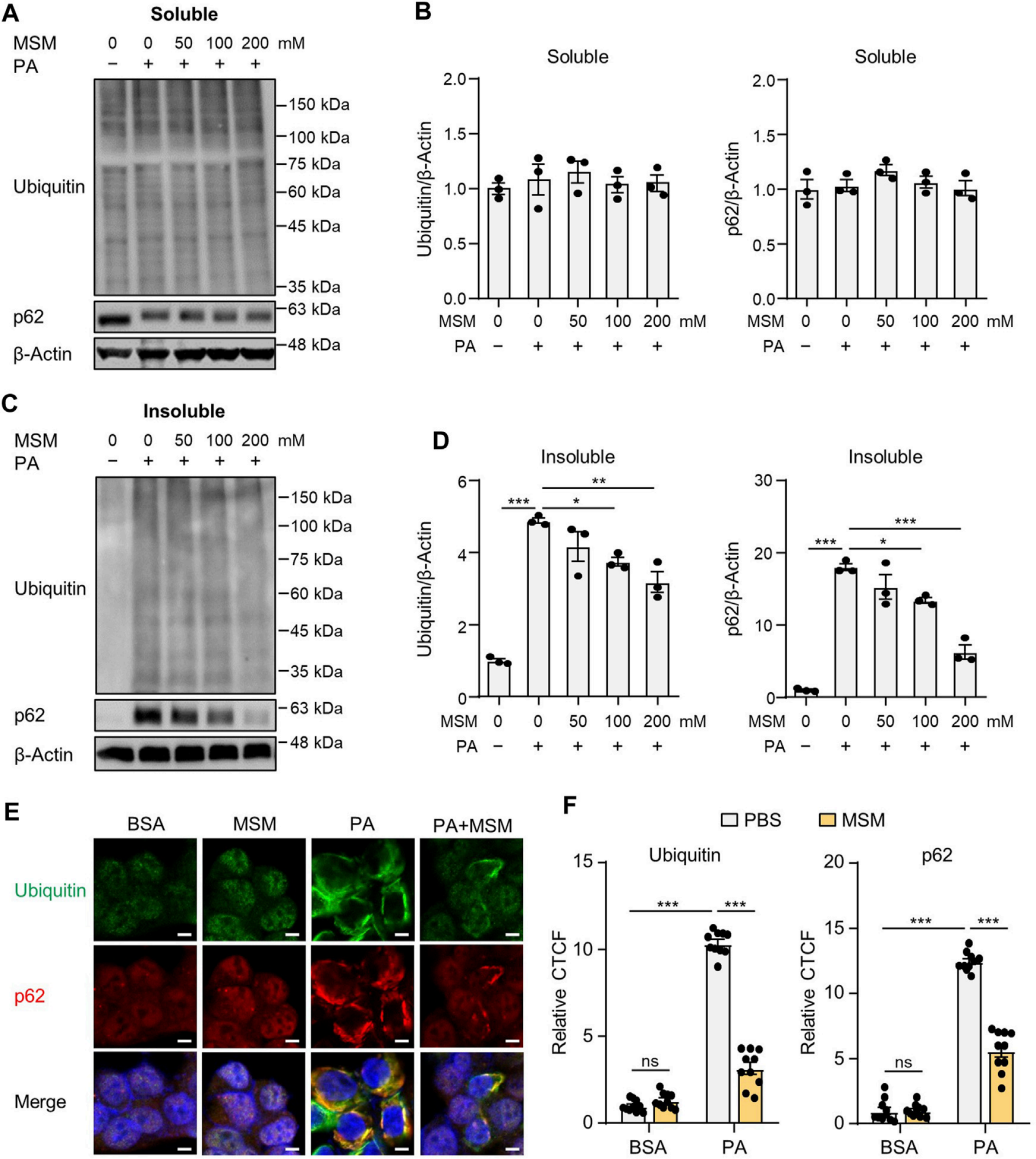
Methylsulfonylmethane (MSM) mitigates palmitic acid (PA)-induced protein aggregation. **(A,C)** Immunoblots for ubiquitin and sequestosome-1 (p62/SQSTM1) from Triton X-100-soluble and -insoluble fractions of HepG2 cells pretreated with the indicated concentrations of MSM (50–200 mM) for 1 h followed by 500 μM PA treatment for 12 h. β-actin was used as a loading control. **(B,D)** Band intensities were quantified and normalized to the control levels. **(E)** Immunofluorescence staining for ubiquitin (green) and p62 (red) in HepG2 cells pretreated with 200 mM MSM for 1 h followed by 500 μM PA treatment for 12 h. Nuclei were stained with 4′,6-diamidino-2-phenylindole (DAPI; blue). Scale bar, 5 μm. **(F)** Images were quantified for corrected total cell fluorescence (CTCF) per unit area. Data are represented as the mean ± standard error of the mean (SEM). *p < 0.05, **p < 0.01, and ***p < 0.001 (one-way **(B,D)** or two-way **(F)** analysis of variance [ANOVA] followed by Tukey’s test).

Saturated fatty acids suppress the autophagic degradation of ubiquitinated proteins *(Park et al., 2014b*; *Lee et al., 2019*; *Kim et al., 2023)*. To investigate the effect of MSM on the autophagic clearance of ubiquitinated proteins in PA-treated HepG2 cells, ubiquitinated protein and p62 levels in detergent-soluble and detergent-insoluble fractions were analyzed via immunoblotting. No significant differences were observed in the levels of ubiquitinated proteins or p62 in the detergent-soluble fractions of HepG2 cells treated with MSM at any dose (Figures 1A, B). However, pretreatment with MSM significantly reduced the levels of ubiquitinated proteins and p62 in the detergent-insoluble fraction of PA-treated HepG2 cells in a dose-dependent manner (Figures 1C, D). To exclude the inhibitory effect of MSM on protein aggregation induced by cytotoxicity, a cell viability test was carried out 12 h after the treatment of HepG2 cells with various doses of MSM (0–400 mM). MSM treatment exerted no cytotoxicity up to 200 mM, whereas significant cytotoxicity was observed at 400 mM (Supplementary Figure S1). Using immunocytochemistry, we further investigated the effects of MSM on the formation of protein aggregates. Pre-treatment with MSM decreased the aggregation of PA-induced ubiquitin- and p62-positive proteins (Figures 1E, F). However, this decrease was not attributed to a reduction in p62 expression (Supplementary Figure S2). These findings suggest that MSM improves the autophagic clearance of ubiquitinated proteins.

### 3.2 MSM prevents PA-induced impairment of autophagic flux

**FIGURE 2:**
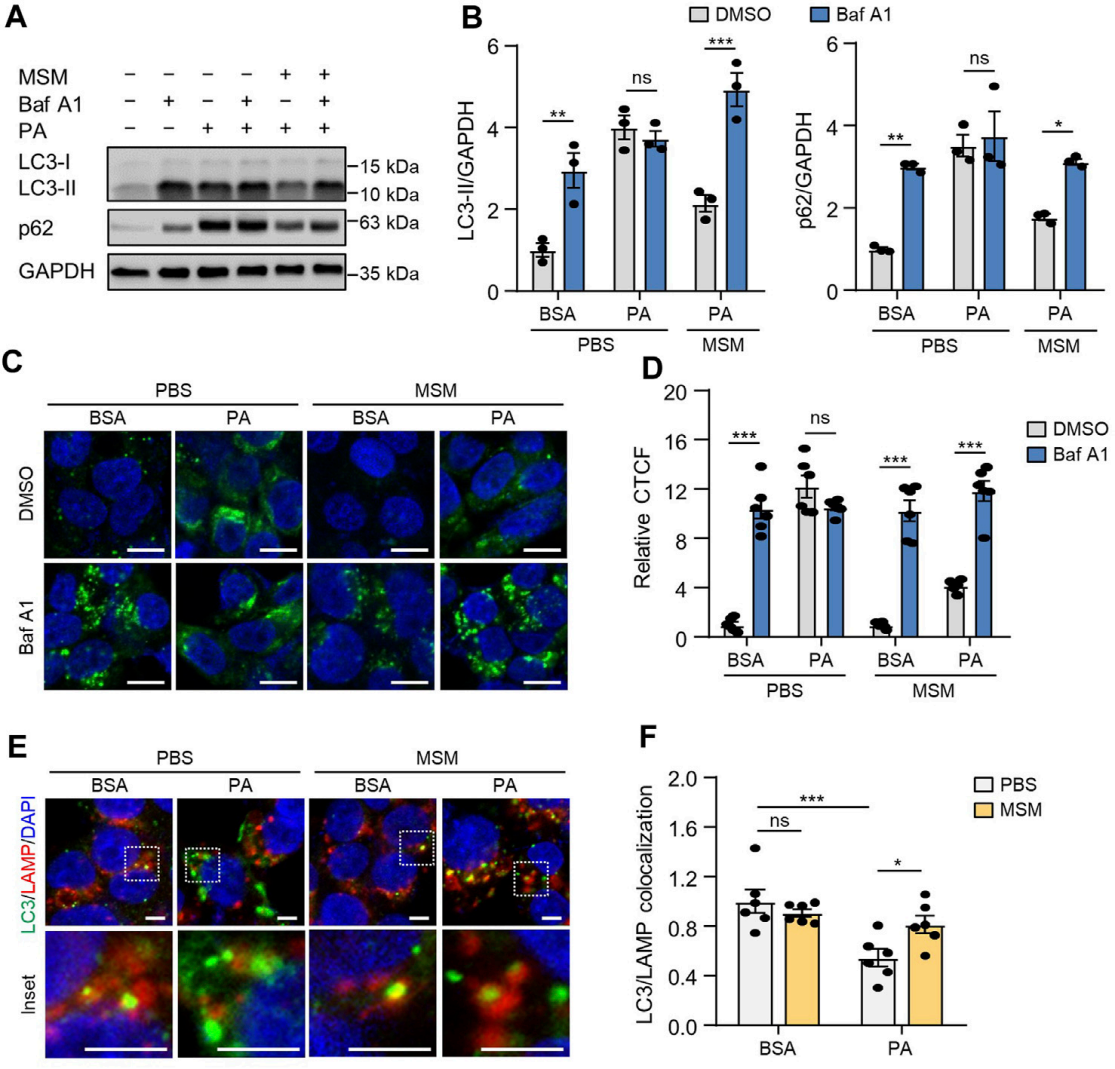
MSM reverses PA-induced impairment of autophagic flux in HepG2 cells. **(A)** Immunoblots of light chain 3 (LC3) and p62 in the cell lysates of HepG2 cells pretreated with 100 mM MSM for 1 h followed by 500 μM PA treatment for 12 h with or without 100 nM bafilomycin A1 for the last 3 h. **(B)** Band intensities were quantified and normalized to the control levels. **(C)** Confocal fluorescence imaging of HepG2 cells transiently transfected with the GFP-LC3 plasmid with the indicated treatments. Nuclei were stained with DAPI (blue). Scale bar, 10 μm. **(D)** Images were quantified for CTCF per unit area. **(E)** Immunofluorescence staining for LC3 (green) and lysosomal-associated membrane protein (LAMP; red) in HepG2 cells with the indicated treatments. Nuclei were stained with DAPI (blue). Boxed areas are magnified in the bottom panels. Scale bar, 5 μm. **(F)** Images were quantified for CTCF per unit area. Data are represented as the mean ± SEM. *p < 0.05, **p < 0.01, and ***p < 0.001 (two-way ANOVA, followed by Tukey’s **(B,D)** or Fisher’s least significant difference [LSD] **(F)** test).

Exposure to PA suppresses autophagic flux in hepatocytes by impairing autophagosome-lysosome fusion (Park et al., 2014b; Park and Lee, 2014). To investigate the effect of MSM on autophagic flux, we assessed MSM-induced autophagic activity using a flux assay based on the immunoblotting of LC3-II and p62. We treated HepG2 cells with bafilomycin A1, a specific inhibitor of vacuolar-type H (+)-ATPase, which inhibits lysosomal enzyme activity and the fusion of autophagosomes with lysosomes. PA-treated cells showed no increase in LC3-II and p62 levels after bafilomycin A1 treatment, whereas MSM pretreatment significantly increased LC3-II and p62 levels in the presence of bafilomycin A1 (Figures 2A, B), suggesting that increased autophagosome degradation, rather than decreased autophagosome formation, was the cause of autophagic degradation by MSM. Confocal fluorescence imaging showed that pretreatment with MSM reduced the number of GFP-LC3 puncta in PA-treated HepG2 cells, whereas treatment with bafilomycin A1 caused a marked accumulation of GFP-LC3 puncta in HepG2 cells co-treated with MSM and PA (Figures 2C, D). To determine whether MSM influenced the fusion of autophagosomes and lysosomes, we analyzed the co-localization of LC3 with the lysosomal marker LAMP-1. Immunofluorescence staining revealed that the colocalization of LC3 puncta with LAMP-1 was significantly reduced in PA-treated HepG2 cells compared to that in control cells, whereas MSM treatment prevented the decrease in PA-treated HepG2 cells (Figures 2E, F). These findings suggest that MSM treatment potentially restores the defective autophagosome–lysosome fusion.

### 3.3 MSM inhibits obesity-induced insulin resistance and hepatic steatosis

**FIGURE 3:**
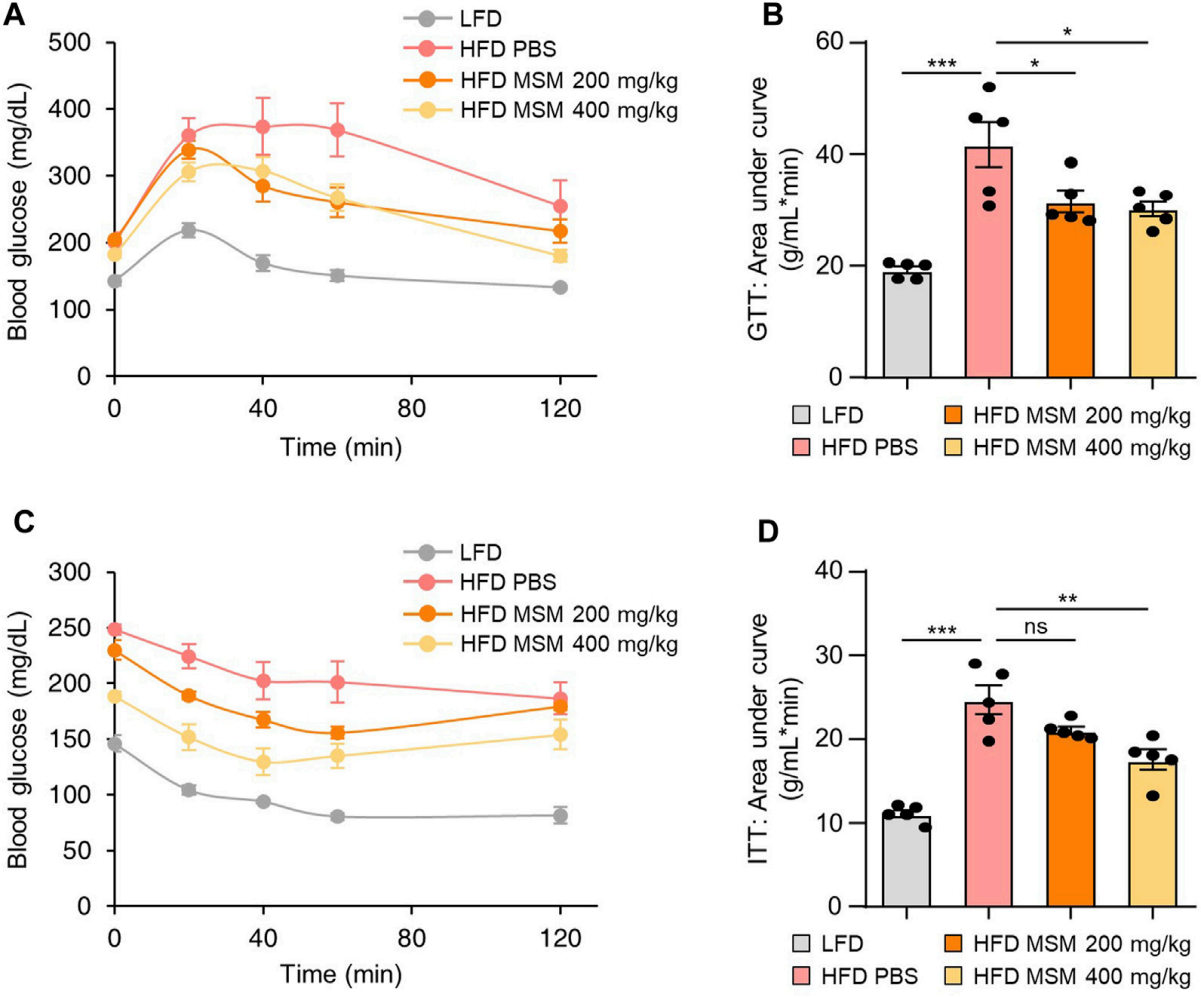
MSM improves the glucose tolerance and insulin sensitivity of mice. **(A–D)** C57BL/6 male mice fed a high-fat diet (HFD) were treated with phosphate-buffered saline (PBS), 200 mg/kg/day p.o. MSM, or 400 mg/kg/day p.o. MSM for 4 weeks. Low-fat diet (LFD)-fed mice of the same age were used as a negative control. Glucose tolerance test (GTT) **(A)** and insulin tolerance test (ITT) **(C)** were conducted using LFD- or HFD-fed mice treated as indicated. The area under the curve was quantified from GTT **(B)** and ITT data **(D)**. Data are represented as the mean ± SEM. *p < 0.05, **p < 0.01, and ***p < 0.001 (one-way ANOVA, followed by Tukey’s test).

To investigate whether oral administration of MSM affected glucose tolerance and insulin sensitivity in HFD-induced obese mice, we fed male mice an HFD or LFD for 9 weeks and gavaged them daily with a vehicle control or 200 or 400 mg/kg body weight MSM for 4 weeks. The glucose tolerance test and calculated area under the curve revealed that MSM-treated HFD-fed mice displayed an elevated clearance rate of blood glucose after injection compared to PBS-treated HFD-fed mice (Figures 3A, B). To determine whether MSM reduces HFD-induced insulin resistance, we conducted a glucose tolerance test in each group. The results revealed that administration of 400 mg/kg MSM significantly enhanced the insulin-mediated glucose-lowering effects (Figures 3C, D). The 200 mg/kg MSM-treated mice also showed improved insulin sensitivity compared to the PBS-treated HFD-fed mice, although the difference was not significant. In lean mice fed LFD, insulin sensitivity and glucose tolerance were unaffected by MSM (Supplementary Figure S3).

**FIGURE 4:**
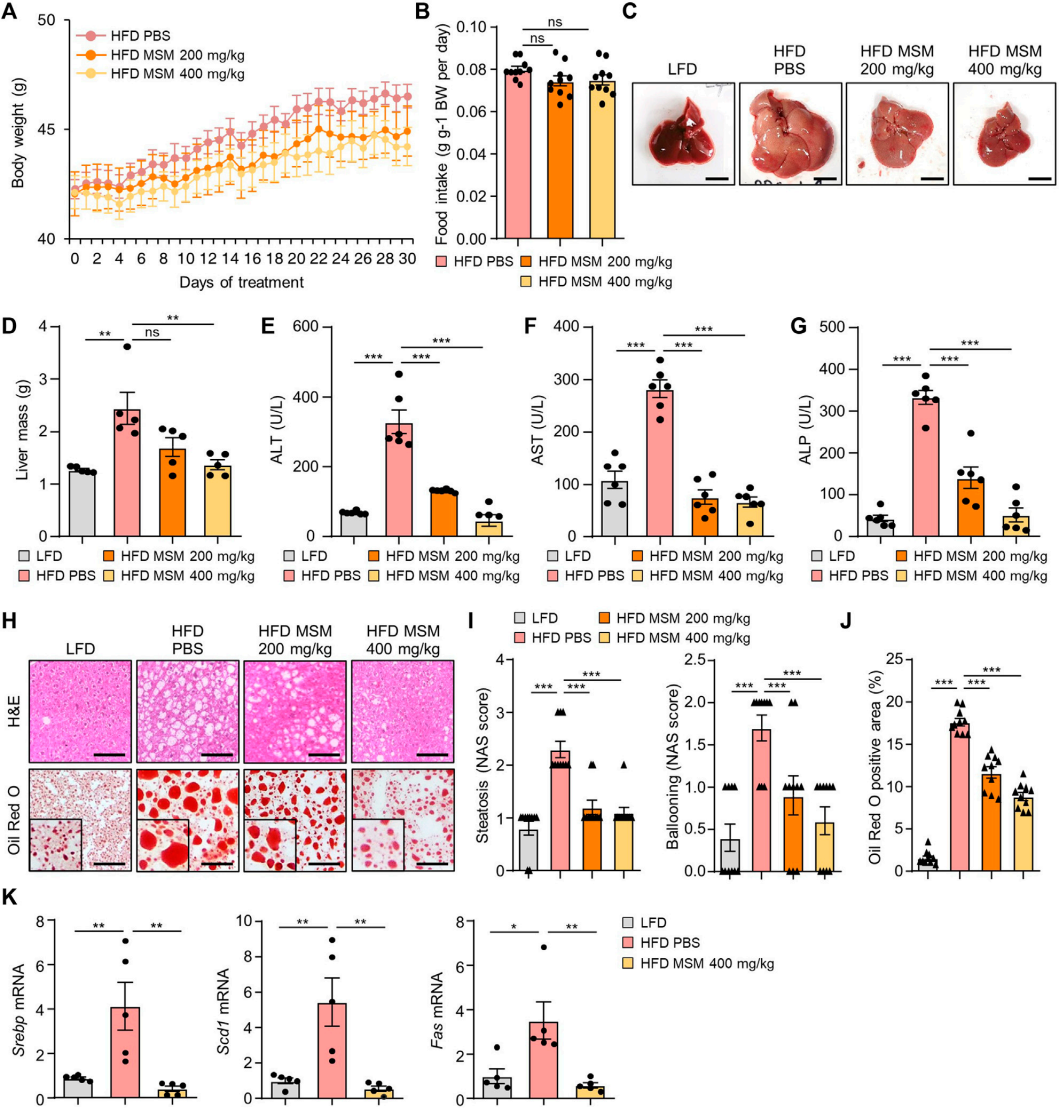
MSM ameliorates HFD-induced obesity and hepatic steatosis. **(A–K)** C57BL/6 male mice fed HFD were treated with PBS, 200 mg/kg/day p.o. MSM, or 400 mg/kg/day p.o. MSM for 4 weeks. LFD-fed mice of the same age were used as a negative control. **(A)** Body weight of mice treated as indicated. **(B)** Food intake during the treatment period. Gross liver morphology **(C)** and total liver mass **(D)** of mice in each group indicated. **(E–G)** Serum alanine aminotransferase (ALT), aspartate aminotransferase (AST), and alkaline phosphatase (ALP) levels. **(H)** Hematoxylin and eosin staining and Oil red O staining of liver sections from mice in each group as indicated. Scale bar, 100 µm. **(I)** Histological non-alcoholic fatty liver disease (NAFLD) activity score (NAS). **(J)** Quantified Oil red O-stained area. **(K)** RT-qPCR analysis of hepatic lipogenic gene expression from mice in each group as indicated. Data are represented as the mean ± SEM. *p < 0.05, **p < 0.01, and ***p < 0.001 (one-way ANOVA, followed by Tukey’s test).

We investigated the effects of MSM on obesity-associated metabolic phenotypes. Treatment of mice with 400 mg/kg significantly reduced the weight gain produced by the HFD (Figure 4A). However, the food intake was not affected by MSM administration (Figure 4B). While the livers of obese mice treated with PBS appeared pale, those of obese mice treated with MSM exhibited a reddish-brown color (Figure 4C). In this model, MSM treatment significantly reduced the liver weight (Figure 4D). Serum levels of ALT, AST, and ALP were significantly lower after administration of MSM (Figures 4E–G). Histological staining of liver tissue sections was performed using hematoxylin and eosin and Oil Red O. PBS-treated obese mice showed signs of hepatocellular ballooning and steatosis, whereas MSM-treated obese mice showed a significant reduction in hepatocellular steatosis and ballooning compared to PBS-treated obese mice (Figures 4H, I). Oil Red O staining revealed a remarkable decrease in hepatic lipid accumulation in MSM-treated obese mice compared to that in PBS-treated obese mice (Figures 4H, J). The expression of genes involved in lipogenesis were reduced in the livers of obese mice treated with MSM (Figure 4K).

### 3.4 MSM attenuates ubiquitinated protein accumulation and inflammation in HFD-induced fatty liver

**FIGURE 5:**
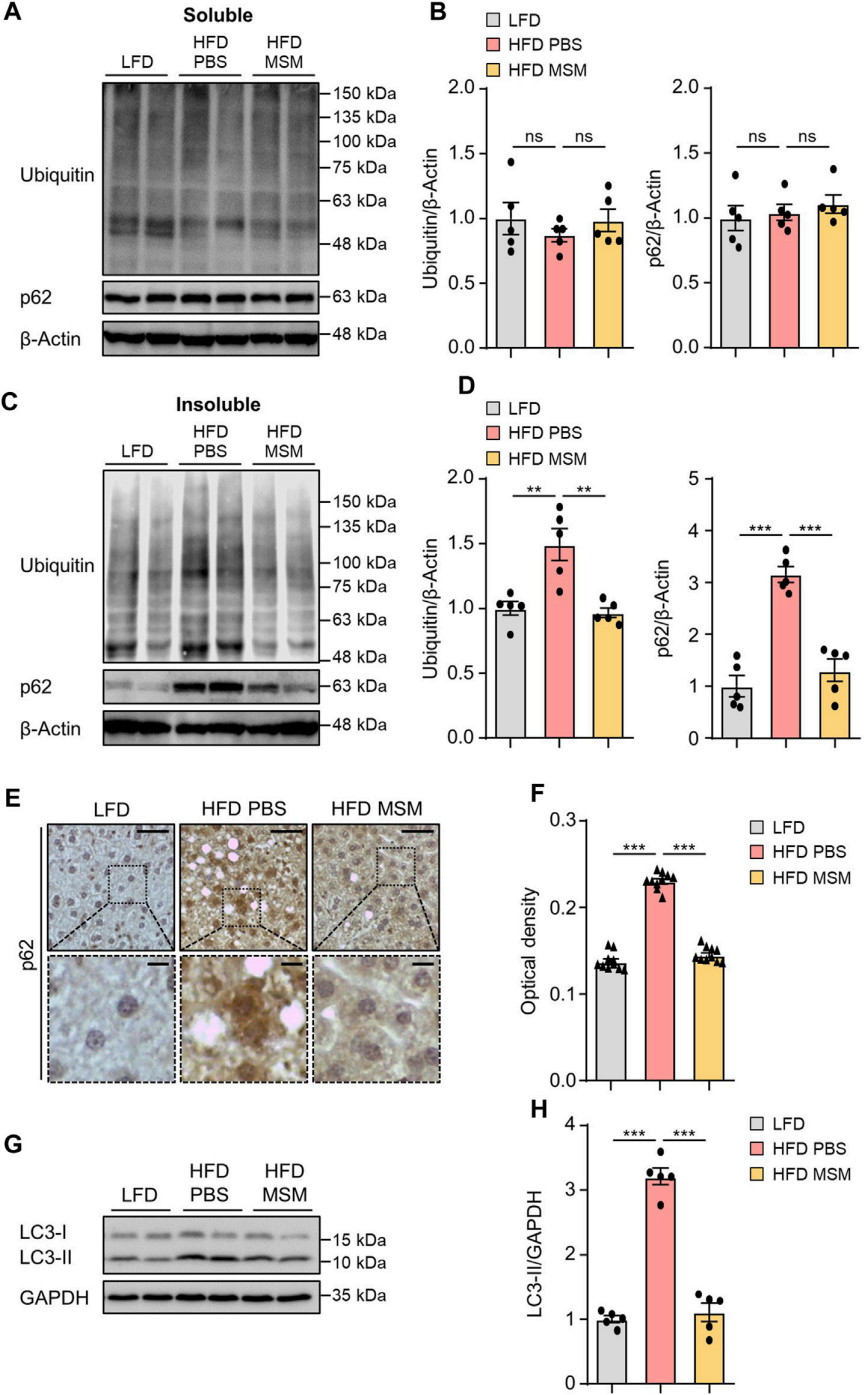
MSM improves the ubiquitinated protein clearance in diet-induced fatty liver. **(A–H)** C57BL/6 male mice fed HFD were treated with PBS or 400 mg/kg/day p.o. MSM for 4 weeks. LFD-fed mice of the same age were used as a negative control. **(A,C)** Immunoblots of ubiquitin and p62 from Triton X-100-soluble and -insoluble fractions of liver tissue lysates. β-actin was used as a loading control. **(B,D)** Band intensities were quantified and normalized to the control levels. **(E)** Immunohistochemical staining for p62 in the liver tissues of mice in each group. Boxed areas are magnified in the bottom panels. Scale bars, 50 and 10 μm (insets). **(F)** Optical density of p62 immunoreactivity. **(G)** Immunoblots of LC3 in the liver tissue lysates of mice in each group. Glyceraldehyde 3-phosphate dehydrogenase (GAPDH) was used as a loading control. **(H)** Band intensities were quantified and normalized to the control levels. Data are represented as the mean ± SEM. *p < 0.05, **p < 0.01, and ***p < 0.001 (one-way ANOVA, followed by Tukey’s test).

We investigated whether administration of MSM influenced autophagy clearance in the livers of HFD-fed mice. Immunoblotting analysis revealed that the levels of ubiquitinated proteins and p62 in the detergent-soluble fractions from liver tissues were not significantly different between PBS- and MSM-treated obese mice (Figures 5A, B). However, MSM administration reduced the elevated levels of ubiquitinated proteins and p62 in the detergent-insoluble fractions of the HFD-fed mouse liver tissues (Figures 5C, D). Immunohistochemical analysis also revealed that the administration of MSM resulted in a significant reduction in p62 staining in the cytoplasm of hepatocytes in HFD-fed mice (Figures 5E, F). Consistent with this, MSM administration significantly alleviated LC3-II protein levels in HFD-fed mouse liver tissues (Figures 5G, H), suggesting that treatment with MSM enhanced the autophagic degradation of ubiquitinated proteins in HFD-induced fatty liver. To evaluate whether MSM administration influenced hepatic inflammation, we measured the expression of inflammatory genes in the livers of lean and obese mice treated with PBS or MSM, using qRT-PCR. Obese mice treated with MSM showed reduced expression of several inflammatory genes, including *Emr1 (also known as F4/80*), *Tnfα*, *Col1a1*, *Tgfβ1*, *Il-1β*, and *Il-6*, compared to PBS-treated obese mice (Supplementary Figure S4).

### 3.5 MSM regulates the AMPK/mTOR/ULK1 signaling pathway

**FIGURE 6:**
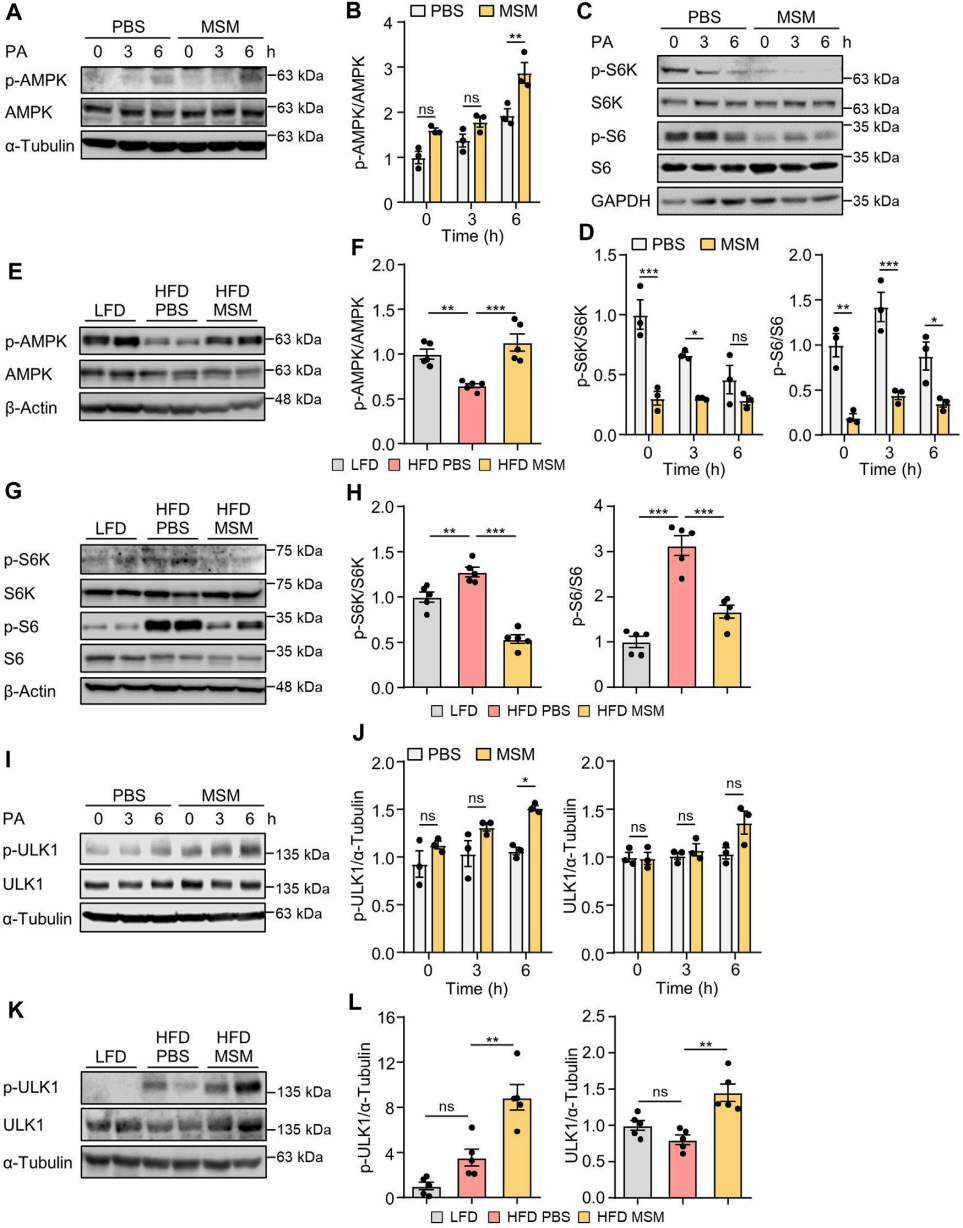
Effects of MSM on the AMP-activated protein kinase (AMPK)/mechanistic target of rapamycin kinase (mTOR)/UNC-51-like autophagy-activating kinase 1 (ULK1) signaling pathway in PA-treated HepG2 cells and in the livers of HFD-fed mice. **(A,C and I)** HepG2 cells were pretreated with 100 mM MSM for 1 h followed by 500 μM PA treatment for the indicated time points. Cell lysates were immunoblotted with the indicated antibodies. GAPDH or α-tubulin served as a loading control. **(B,D and J)** Band intensities were quantified and normalized to the control levels. **(E,G and K)** C57BL/6 male mice fed HFD were treated with PBS or 400 mg/kg/day p.o. MSM for 4 weeks. LFD-fed mice of the same age were used as a negative control. Liver tissue lysates were immunoblotted with the indicated antibodies. β-actin or α-tubulin served as a loading control. **(F, H, and L)** Band intensities were quantified and normalized to the control levels. Data are represented as the mean ± SEM. *p < 0.05, **p < 0.01, and ***p < 0.001 (one-way ANOVA, followed by Tukey’s test).

AMPK positively regulates autophagy by detaching mTORC1 from the ULK1 complex (Salminen and Kaarniranta, 2012). To explore the potential mechanisms by which MSM enhances autophagy, we investigated the signaling pathways involving AMPK and mTORC1, two key upstream signaling molecules responsible for regulating autophagy. Immunoblotting analysis revealed increased phosphorylation of AMPK (Supplementary Figures S5A–D) and decreased phosphorylation of p70S6K (Supplementary Figures S6A–D) in HepG2 cells by MSM in a dose- and time-dependent manner. Next, we examined the effects of MSM on the activities of AMPK and mTORC1 in PA-treated HepG2 cells and livers of HFD-fed mice. MSM treatment increased AMPK activation (Figures 6A, B) but suppressed the phosphorylation of p70S6K and ribosomal protein S6 in PA-treated HepG2 cells (Figures 6C, D). Correspondingly, obese mice treated with MSM showed a significant increase in phosphorylated AMPK levels (Figures 6E, F) and a reduction in the levels of phosphorylated p70S6K and ribosomal protein S6 compared to those in PBS-treated obese mice (Figures 6G, H). To further determine whether ULK1 signaling is involved in MSM attenuation of impaired autophagy in MAFLD, the phosphorylation of ULK1 at Serine-555 (S555) was assessed. We observed a dose- and time-dependent increase in ULK1 S555 phosphorylation in MSM-treated HepG2 cells (Supplementary Figures S7A–D). Immunoblotting analysis revealed that MSM treatment enhanced ULK1 S555 phosphorylation in PA-treated HepG2 cells (Figures 6I, J). Consistently, ULK1 S555phosphorylation was significantly higher in the livers of obese mice treated with MSM than in those treated with PBS (Figures 6K, L). Collectively, these data suggest that the AMPK/mTOR/ULK1 signaling pathway is involved in MSM-mediated enhancement of hepatic autophagy.

### 3.6 MSM enhances autophagy via an AMPK/mTORC1-dependent pathway

**FIGURE 7:**
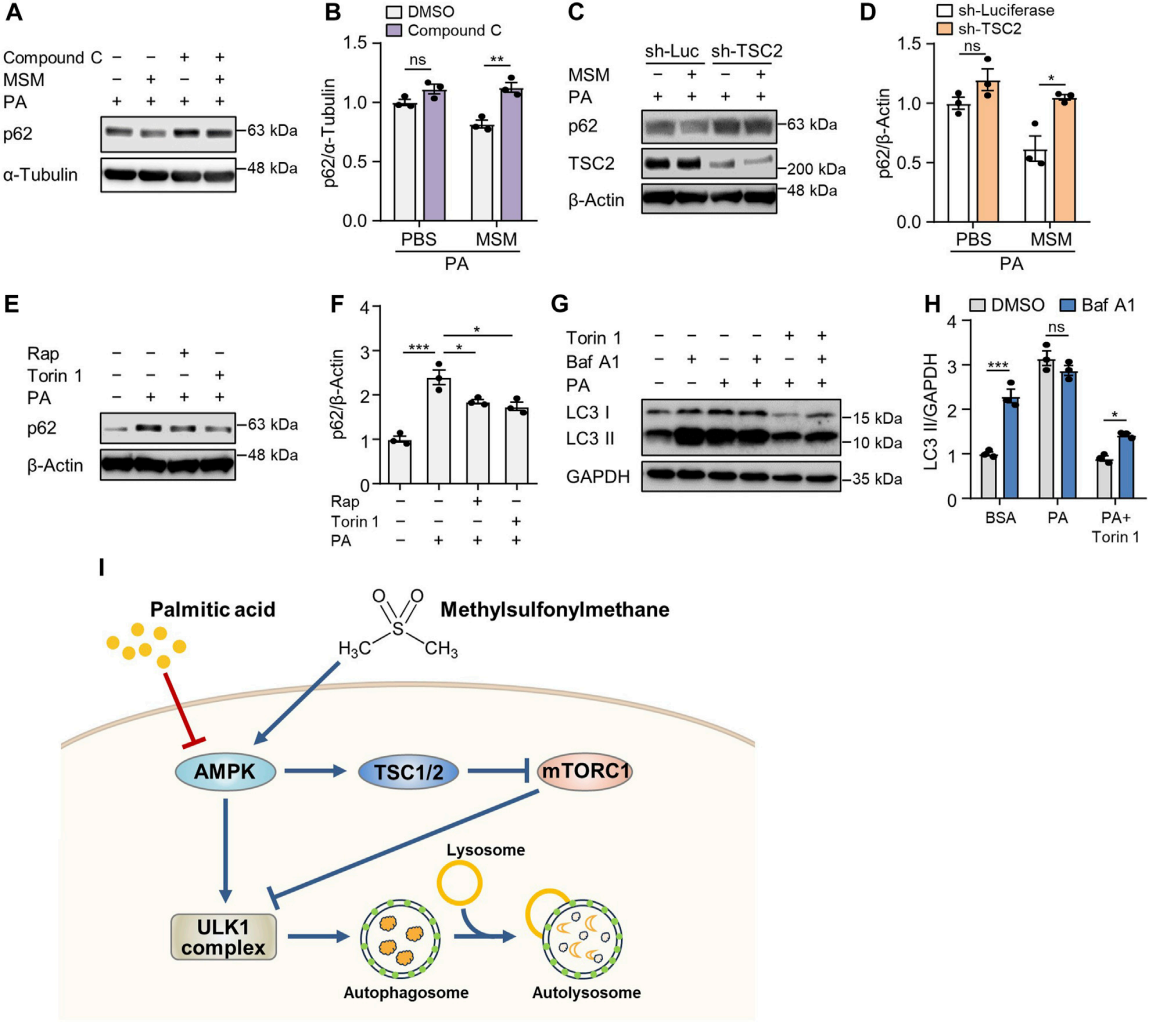
MSM enhances autophagy by regulating the AMPK/mTOR signaling pathway. **(A)** Immunoblots of p62 in the cell lysates of HepG2 cells pretreated with 20 µM compound C or 100 mM MSM for 30 min followed by 500 μM PA treatment for 9 h. **(C)** Immunoblots of p62 and TSC2 in the cell lysates of lentiviral sh-Luc- or sh-TSC2-infected HepG2 cells pretreated with 100 mM MSM for 30 min followed by 500 μM PA treatment for 9 h. **(E)** Immunoblots of p62 in the cell lysates of HepG2 cells pretreated with 100 nM rapamycin or 250 nM Torin 1 for 30 min followed by 500 μM PA treatment for 9 h. **(G)** Immunoblots of LC3 and p62 in the cell lysates of HepG2 cells pretreated with 250 nM Torin 1 for 1 h followed by 500 μM PA treatment for 12 h with or without bafilomycin A1 (100 nM) for the last 3 h **(B,D,F and H)** Band intensities were quantified and normalized to the control levels. Data are represented as the mean ± SEM. *p < 0.05, **p < 0.01, and ***p < 0.001 (one-way ANOVA, followed by Tukey’s test). **(I)** Schematic diagram of the underlying mechanism of the protective effects of MSM against obesity-induced metabolic dysregulation in hepatocytes.

To determine whether AMPK signaling in MSM-treated cells influences the autophagic degradation of p62, we pretreated HepG2 cells with the AMPKα inhibitor compound C. Immunoblotting analysis revealed that pretreatment with compound C abrogated MSM-induced reduction of p62 levels in PA-treated cells (Figures 7A, B). Next, we investigated whether the enhancement of autophagy by MSM treatment was associated with mTORC1 signaling. We found that the lentiviral knockdown of TSC2 did not have any significant effect on MSM-induced autophagic degradation in PA-treated HepG2 cells (Figures 7C, D). Consistent with the inhibitory effect of MSM on mTORC1 signaling, the mTOR inhibitors, rapamycin, and Torin 1, significantly reduced the accumulation of p62 in PA-treated cells (Figures 7E, F). Next, we monitored the autophagic flux by analyzing the LC3 turnover in HepG2 cells cotreated with Torin 1 and PA. In the presence of Torin 1 and PA, HepG2 cells treated with bafilomycin A1 had higher expression of LC3-II than those without bafilomycin A1 treatment (Figures 7G, H). Collectively, these data suggest that MSM-enhanced hepatic autophagy is mediated by the AMPK/mTOR signaling pathway.

## 4 Discussion

Owing to the increasing prevalence of obesity and metabolic syndrome, MAFLD has emerged as the most common chronic liver ailment in both industrialized and developing nations (Adams et al., 2020). No specific medicine can suppress the progression of MAFLD, and existing drugs with therapeutic effects have many limitations. Several natural compounds have been shown to exert therapeutic effects against MAFLD. Although MSM holds promise as a potential therapeutic modality for mitigating obesity-associated metabolic disorders, including type 2 diabetes and fatty liver disease (Sousa-Lima et al., 2016; Miller et al., 2021), the precise underlying mechanism by which it exerts its beneficial effects on obesity-induced metabolic perturbations remains unclear. In this study, we investigated the potential therapeutic effects of MSM on MAFLD and the underlying mechanisms involving autophagic flux via the AMPK/mTOR/ULK1 signaling pathway. We found that MSM treatment effectively ameliorated MAFLD-associated pathological changes. Moreover, obesity-induced insulin resistance, hepatic steatosis, and inflammation were noticeably attenuated in the MSM-treated group than in the vehicle-treated control group. These findings are consistent with those of previous studies showing the beneficial effects of MSM on various metabolic and inflammatory disorders (Ahn et al., 2015; Sousa-Lima et al., 2016; Miller et al., 2021). The present study further expands these observations by demonstrating the potential of MSM to mitigate MAFLD progression.

MSM has gained significant attention as a dietary supplement because of its potential therapeutic effects on various health conditions, primarily those related to joint health and inflammation (Kim et al., 2009; Nakhostin-Roohi et al., 2011). MSM is generally well tolerated and recognized as safe by the FDA. Other studies have demonstrated the beneficial impact of sulfur-containing amino acids, such as methionine, cysteine, and taurine, on glucose homeostasis in patients with diabetes and animal models (Ribeiro et al., 2009; Manna et al., 2013; Dahlhoff et al., 2014; Xu et al., 2015). Owing to the presence of two methyl groups within their structure, MSM potentially function as a source of methyl donation, thereby mitigating hepatic steatosis (Sousa-Lima et al., 2016). Interestingly, methyl donors have been shown to mitigate hepatic lipid accumulation in numerous nutritional models (Song et al., 2007; Kwon et al., 2009; Wang et al., 2010). Consistent with this finding, we found that hepatocellular ballooning and steatosis decreased in MSM-treated obese mice in this study. Solubility is a crucial factor influencing the bioavailability and efficacy of MSM. MSM exhibits high solubility in aqueous solutions in the neutral to slightly alkaline pH range, facilitating its absorption and potential therapeutic action (Kaiser et al., 2020). Therefore, consideration of the formulation and administration methods of MSM is important to optimize its solubility and subsequent absorption. Its reported adverse effects, including gastrointestinal discomfort, bloating, and headache, are typically mild (Liu et al., 2018; Crawford et al., 2019). However, the limited long-term safety data on MSM necessitates further research to facilitate its long-term use.

Autophagy plays a crucial role in maintaining cellular homeostasis by regulating the turnover of organelles and various macromolecules, including proteins and lipids (Choi et al., 2013; Gatica et al., 2018). In various liver diseases, impaired autophagic flux is involved in the accumulation of lipid droplets in hepatocytes, progression of hepatic steatosis, and hepatic inflammation (Park et al., 2014b; Kim et al., 2018; Niture et al., 2021). We and other researchers have previously reported that increased autophagic activity enhances the metabolic profile of mice with metabolic syndrome and obesity (Park and Lee, 2014; Lim et al., 2018; Kim et al., 2023). In the present study, MSM restored the autophagic flux in PA-treated HepG2 cells and HFD-fed mouse liver tissues. The observed reduction in ubiquitinated protein and p62 levels in the detergent-insoluble fractions of cells and liver tissues in MSM-treated group suggests that MSM promotes autophagic degradation in MAFLD. This effect is likely to contribute to a reduction in lipid accumulation and an overall improvement in hepatic function.

AMPK/mTOR/ULK1 signaling pathway is a well-established regulator of autophagy. AMPK, a highly conserved serine/threonine-protein kinase throughout evolution, serves as a key energy sensor regulating metabolic processes and inflammatory responses (Paquette et al., 2021). AMPK directly phosphorylates several components of the mTORC1 pathway, leading to the downregulation of mTORC1 activity and subsequent promotion of cellular autophagy (Inoki et al., 2003; Holczer et al., 2019). Autophagy initiation is also hindered by mTOR activation via the regulation of ULK1 ubiquitylation (Nazio et al., 2013). Furthermore, autophagy is enhanced by the suppression of the activation of p70S6K, a pivotal kinase downstream of mTOR (Sun et al., 2018). Here, phosphorylated AMPK level was increased, whereas phosphorylated p70S6K and ribosomal protein S6 levels were reduced by MSM treatment (Figure 7I). Moreover, compound C, an inhibitor of AMPKα, abrogated MSM-induced autophagy in PA-treated HepG2 cells. Consistent with this finding, lentiviral knockdown of *TSC2* did not significantly affect the autophagic degradation promoted by MSM treatment in HepG2 cells exposed to PA. Therefore, the correlation between MSM-induced autophagy and the AMPK/mTOR axis suggests that the beneficial effects of MSM may be due to its ability to enhance autophagy via the AMPK/mTOR/ULK1 pathway.

Despite the promising findings, our study has limitations. First, our study was conducted on mice, so it is unclear whether the same results would be observed in humans. Future research should use clinical trials to validate the therapeutic potential of MSM in a more physiologically relevant context. Additionally, investigating the long-term effects of MSM on metabolic parameters and liver function will be crucial for establishing its clinical relevance. Second, the study did not explore the possibility that other mechanisms, including inflammation, may be involved in the improved insulin sensitivity caused by MSM treatment. Cytokines secreted by activated Kupffer cells and adipocytes can further exacerbate inflammation and insulin resistance (de Luca and Olefsky, 2008). Therefore, understanding the specific interactions among inflammation, insulin resistance, and defective autophagy will provide deeper insights into the mechanisms of action.

## 5 Conclusion

Taken together, this study highlights the beneficial effects of MSM in ameliorating MAFLD by restoring the autophagic flux via the AMPK/mTOR/ULK1 signaling pathway. These findings provide a mechanistic basis for the observed improvements in obesity-induced insulin resistance and hepatic steatosis. The potential translational implications of our findings further highlight the importance of exploring the therapeutic potential of MSM in MAFLD and related metabolic disorders.

## Data Availability

The raw data supporting the conclusion of this article will be made available by the authors, without undue reservation.
