# Supplementary material for: Methylsulfonylmethane ameliorates metabolic-associated fatty liver disease by restoring autophagy flux via AMPK/mTOR/ULK1 signaling pathway
Source: Front Pharmacol. 2023 Nov 30;14:1302227. doi: 10.3389/fphar.2023.1302227 (PMC10720622; doi:10.3389/fphar.2023.1302227)
Supplement: Supplementary file 1 [file Table1.DOCX]

Supplementary Material

Methylsulfonylmethane ameliorates metabolic-associated fatty liver disease by restoring autophagy flux via AMPK/mTOR/ULK1 signaling pathway


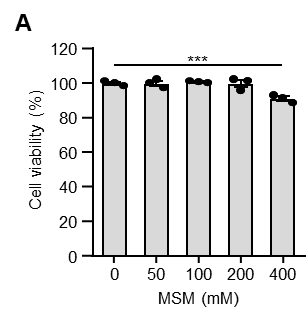


**Supplementary Figure 1.** In vitro cytotoxicity of methylsulfonylmethane (MSM) in HepG2 cells. HepG2 cells were treated with the indicated concentration of MSM for 9 h. Cell viability was measured using the water-soluble tetrazolium salt (WST)-8 assay. Data are represented as the mean ± standard error of the mean (SEM). ***p < 0.001 (one-way analysis of variance [ANOVA], followed by Tukey’s test).


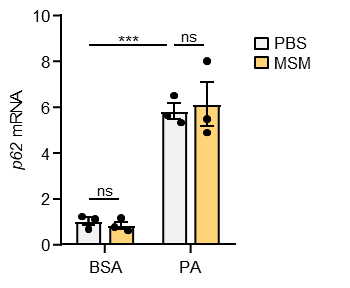


**Supplementary Figure 2.** Reverse transcription-quantitative polymerase chain reaction (RT-qPCR) analysis of *p62* mRNA levels in HepG2 cells pretreated with 100 mM MSM for 30 min followed by 500 μM PA treatment for 9 h. Data are represented as the mean ± SEM. ***p < 0.001 (two-way ANOVA, followed by Tukey’s test).


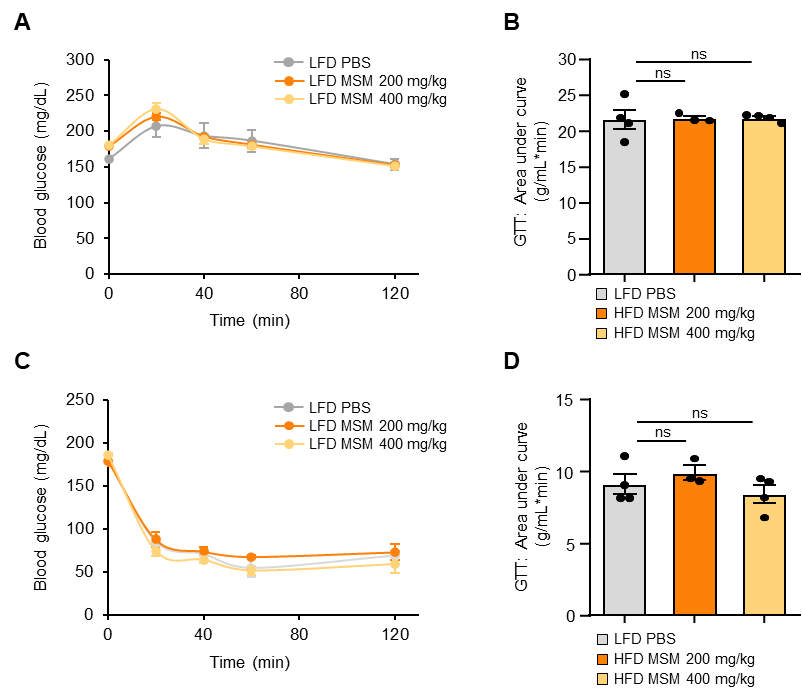


**Supplementary Figure 3.** MSM treatment does not affect insulin sensitivity and glucose tolerance in lean mice. (A-D) C57BL/6 male mice fed a low-fat diet (LFD) were treated with phosphate-buffered saline (PBS), 200 mg/kg/day p.o. MSM, or 400 mg/kg/day p.o. MSM for four weeks. Glucose tolerance test (GTT, A) and insulin tolerance test (ITT, C) were conducted using LFD- or HFD-fed mice treated as indicated. The area under the curve was quantified from GTT (B) and ITT data (D). Data are represented as the mean ± SEM. *p < 0.05, **p < 0.01, and ***p < 0.001 (one-way ANOVA, followed by Tukey’s test).


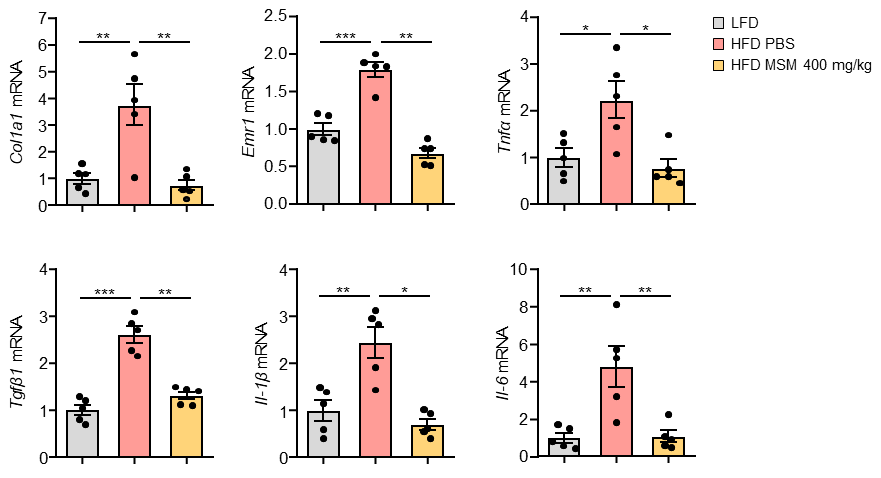


**Supplementary Figure 4.** RT-qPCR analysis of inflammatory gene expression in the livers of lean and obese mice treated with phosphate-buffered saline (PBS) or MSM. C57BL/6 male mice fed HFD were treated with PBS or 400 mg/kg/day p.o. MSM for 4 weeks. Low-fat diet (LFD)-fed mice of the same age were used as a negative control. Liver tissues were collected from mice and analyzed via RT-qPCR to determine the expression levels of *Emr1*, *Tnfα*, *Col1a1*, *Tgfβ1*, *Il-1β*, and *Il-6*. Data are represented as the mean ± SEM. **p < 0.01 and ***p < 0.001 (one-way ANOVA, followed by Tukey’s test).


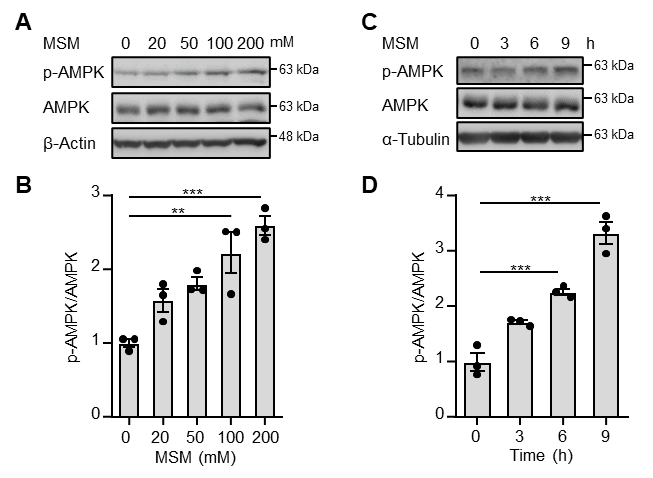


**Supplementary Figure 5.** Dose- and time-dependent effects of MSM on AMP-activated protein kinase (AMPK) phosphorylation. (A) HepG2 cells were treated with the indicated concentrations of MSM (20–200 mM) for 9 h. Cell lysates were immunoblotted with the indicated antibodies. (C) HepG2 cells were treated with 200 mM MSM for the indicted time points. Cell lysates were immunoblotted with the indicated antibodies. β-actin or α-tubulin served as a loading control. (B, D) Band intensities were quantified and normalized to the control levels. Data are represented as the mean ± SEM. **p < 0.01 and ***p < 0.001 (one-way ANOVA, followed by Tukey’s test).

**
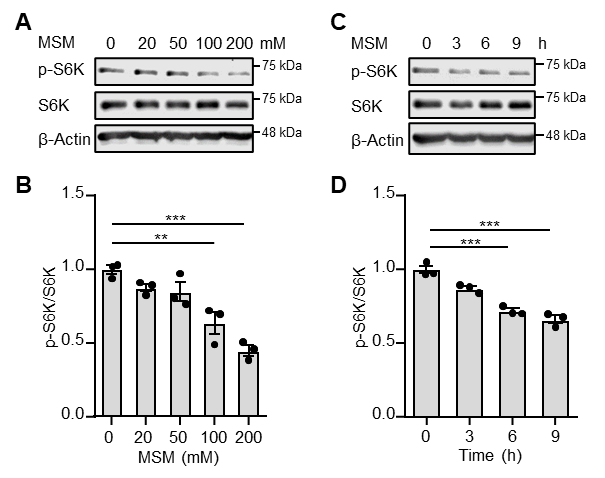
**

**Supplementary Figure 6.** Dose- and time-dependent effects of MSM on p70S6K phosphorylation. (A) HepG2 cells were treated with the indicated concentrations of MSM (20–200 mM) for 9 h. Cell lysates were immunoblotted with the indicated antibodies. (C) HepG2 cells were treated with 200 mM MSM for the indicted time points. Cell lysates were immunoblotted with the indicated antibodies. β-actin or α-tubulin served as a loading control. (B, D) Band intensities were quantified and normalized to the control levels. Data are represented as the mean ± SEM. **p < 0.01 and ***p < 0.001 (one-way ANOVA, followed by Tukey’s test).


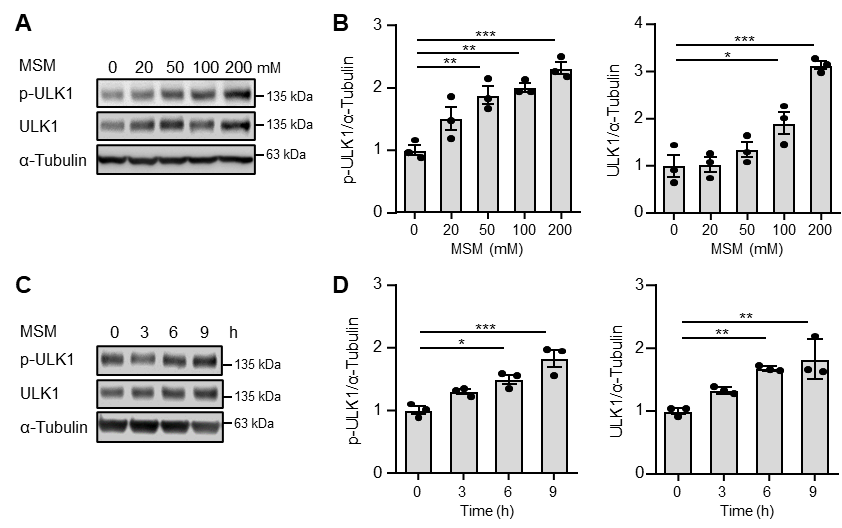


**Supplementary Figure 7.** Dose- and time-dependent effects of MSM on total and phosphorylated UNC-51-like autophagy-activating kinase 1 (ULK1). (A) HepG2 cells were treated with the indicated concentrations of MSM (20–200 mM) for 9 h. Cell lysates were immunoblotted with the indicated antibodies. (C) HepG2 cells were treated with 200 mM MSM for the indicted time points. Cell lysates were immunoblotted with the indicated antibodies. β-actin or α-tubulin served as a loading control. (B, D) Band intensities were quantified and normalized to the control levels. Data are represented as the mean ± SEM. **p < 0.01 and ***p < 0.001 (one-way ANOVA, followed by Tukey’s test).

**Supplementary Table1.** List of primer pairs used for qRT-PCR analysis.

| **Gene** | **Forward Primer (5’-3’)** | **Reverse Primer (5’-3’)** |
| --- | --- | --- |
| mouse Cyclophilin A | GAGCTGTTTGCAGACAAAGTTC | CCCTGGCACATGAATCCTGG |
| mouse Tnfα | TCCCAGGTTCTCTTCAAGGGA | GGTGAGGAGCACGTAGTCGG |
| mouse Tgfβ1 | CTCCCGTGGCTTCTAGTGC | GCCTTAGTTTGGACAGGATCTG |
| mouse Col1a1 | GCTCCTCTTAGGGGCCACT | CCACGTCTCACCATTGGGG |
| mouse Emr1 | CCCCAGTGTCCTTACAGAGTG | GTGCCCAGAGTGGATGTCT |
| mouse Il-1β | TCTTTGAAGTTGACGGACCCCC | TGAGTGATACTGCCTGCCTG |
| mouse Il-6 | TAGTCCTTCCTACCCCAATTTCC | TTGGTCCTTAGCCACTCCTTC |
| mouse Srebp-1c | CGGAAGCTGTCGGGGTAG | GTTGTTGATGAGCTGGAGCA |
| mouse Scd1 | GAGGCCTGTACGGGATCA | GCCCAGTCGTACACGTCA |
| mouse Fas | TGGGTTCTAGCCAGCAGAGT | ACCACCAGAGACCGTTATGC |
| human Cyclophilin A | GCAAAGTGAAAGAAGGCATGAA | CCATTCCTGGACCCAAAGC |
| human p62 | CCGCCGCTTCAGCTTCTGCT | GTTCCCGCCGGCACTCCTTC |
